# Astragalosides Supplementation Enhances Intrinsic Muscle Repair Capacity Following Eccentric Exercise-Induced Injury

**DOI:** 10.3390/nu14204339

**Published:** 2022-10-17

**Authors:** Tzu-Shao Yeh, Tze-Huan Lei, Matthew J. Barnes, Lei Zhang

**Affiliations:** 1 School of Public Health, Nantong University, Nantong 226019, China; 2 Institute of Interdisciplinary Integrative Medicine Research, School of Medicine, Nantong University, Nantong 226001, China; 3 College of Physical Education, Hubei Normal University, Huangshi 435002, China; 4 School of Sport, Exercise and Nutrition, Massey University, Palmerston North 4410, New Zealand

**Keywords:** astragalus, inflammatory, muscle damage, muscular strength, delayed-onset muscle soreness, muscle regeneration, traditional Chinese medicine

## Abstract

Astragalosides have been shown to enhance endurance exercise capacity in vivo and promote muscular hypertrophy in vitro. However, it remains unknown whether astragalosides supplementation can alter inflammatory response and enhance muscle recovery after damage in humans. We therefore aimed to evaluate the effect of astragalosides supplementation on muscle’s intrinsic capacity to regenerate and repair itself after exercise-induced damage. Using a randomized double-blind placebo-controlled cross-over design, eleven male participants underwent 7 days of astragalosides supplementation (in total containing 4 mg of astragalosides per day) or a placebo control, following an eccentric exercise protocol. Serum blood samples and variables related to muscle function were collected prior to and immediately following the muscle damage protocol and also at 2 h, and 1, 2, 3, 5, and 7 days of the recovery period, to assess the pro-inflammatory cytokine response, the secretion of muscle regenerative factors, and muscular strength. Astragalosides supplementation reduced biomarkers of skeletal muscle damage (serum CK, LDH, and Mb), when compared to the placebo, at 1, 2, and 3 days following the muscle damage protocol. Astragalosides supplementation suppressed the secretion of IL-6 and TNF-α, whilst increasing the release of IGF-1 during the initial stages of muscle recovery. Furthermore, following astragaloside supplementation, muscular strength returned to baseline 2 days earlier than the placebo. Astragalosides supplementation shortens the duration of inflammation, enhances the regeneration process and restores muscle strength following eccentric exercise-induced injury.

## 1. Introduction

Muscle repair and myogenesis are tightly regulated by a finely controlled inflammatory response. A transient increase in local inflammatory signaling is now believed to be an important process of skeletal muscle repair and remodeling [1]. However, chronic persistence of intramuscular inflammation could potentially lengthen the recovery period, as well as resulting in maladaptation of the skeletal muscles.

Injured muscle fibers and local inflammatory responses act as stress signals to trigger pro-myogenic signaling and multiple intracellular signaling cascades, such as IGFs-PI3K-Akt-mTOR cascade and Raf-MAPK-Erk cascade, during the initial phase of muscle injury. In addition, Schertzer et al. [2] revealed that local insulin-like growth factors (IGFs) are up-regulators of these cascades, which increase significantly after muscle injury. Amongst those IGFs, overexpression of IGF-I accelerates the regenerative process of injured skeletal muscles. Previous studies suggested that IGF-I activates satellite cells and is involved with myoblast proliferation and differentiation, ultimately accelerating the regenerative process of injured skeletal muscles [3,4]. In contrast, faster clearance of the local pro-inflammatory cytokines, such as interleukin-1 (IL-1), interleukin-6 (IL-6), and tumor necrosis factor-alpha (TNF-α) can reduce local skeletal muscle pain following muscle injuries [5]. Furthermore, reduced inflammatory response after strenuous exercise and elimination of the accumulation of neutrophils located inside the skeletal muscle vasculature are important indicators of muscle recovery after exercise [6]. Consequently, avoiding prolonged intramuscular inflammation can alleviate the symptoms of muscle damage, and thus result in timely muscle regeneration, with faster restoration of the skeletal muscle-tissue ultrastructure.

The notion that inflammation is a crucial process for muscular repair and regeneration is now gaining acceptance, and studies have suggested that in addition to intramuscular cytokines, changes in and recovery of muscle force-generating capacity should be considered as the objective indication of muscle recovery after injury [7,8]. Blood flow to skeletal muscle increases during exercise to match exercise oxygen demand and to remove metabolic byproducts [9]. However, overload-induced muscle injuries that cause myofilament disruption, sarcolemma breakdown, and muscle protein leakage could subsequently result in reduced oxygen saturation inside the skeletal muscle [10]. Several natural compounds with potent bioactive antioxidant and anti-inflammatory properties are currently used as nutraceuticals and supplements to maintain skeletal muscle health [11,12].

*Astragalus membranaceus* is one of the most popular herbal tonics in many Asian countries. Astragalosides are biologically active substances from the plant *Astragalus*, that could protect against CCl4-induced acute liver injury [13], PQ-induced lung injury [14], or IS-induced kidney injury [15] in mice or rats by ameliorating oxidative stress. Our previous study revealed that *Astragalus membranaceus* is a nutritional activator that promotes myogenesis by stimulating Akt/mTOR signaling in skeletal muscle cell lines [16] and can stabilize the sarcolemma, as well as preventing exercise-induced fatigue in rodents [17]. Although the wide use of astragalus as an antioxidant and anti-inflammatory agent has been well demonstrated, no research has been conducted on its efficacy in preventing prolonged inflammation and muscle repair following eccentric exercise-induced muscle damage. Furthermore, no studies are available to observe whether astragalosides supplementation could potentially alter the initial phase of muscle damage and whether this can result in faster skeletal muscle regeneration and recovery of muscular strength following eccentric exercise.

For this purpose, we conducted a randomized double-blind placebo-controlled cross-over study on healthy young men, to investigate the effects of astragalosides on the time course of skeletal muscle recovery after eccentric exercise-induced injury. We previously demonstrated the ability of astragalosides to facilitate better myotube regeneration and skeletal muscle hypertrophy in vitro [16], and therefore hypothesized that astragalosides supplementation would alter the initial muscle damage response and promote faster skeletal muscle repair and recovery of muscular strength following eccentric exercise-induced muscle damage.

## 2. Materials and Methods

### 2.1. Subjects

Twelve recreationally active, healthy male adults, with no history of regular resistance training participated in this study. One subject dropped out during the study because of an acute back problem. All subjects had no previous sports injuries, were free from metabolic disorders or diseases, had no history of anabolic steroid use, and had abstained from taking any thyroid, hyperlipidmeic, hypoglycemic, or androgenic medication, or other purported anabolic or ergogenic nutritional supplements for two months before beginning the study. All subjects signed informed consent documents. The study was approved by the Human Research Ethics Committee of the National Taiwan Sport University (Taoyuan, Taiwan) and conformed to the declaration of Helsinki.

### 2.2. Muscle Damage Protocol

All subjects underwent an eccentric exercise protocol to induce muscle damage in the quadriceps of one leg. This eccentric exercise protocol consisted of 5 sets of 10 repetitions of unilateral leg extensions, with a load corresponding to the 120% of the subject’s predicted one repetition maximum (1 RM); each set was separated by a 3 min rest interval. Each repetition was completed over 3 s, controlled by the metronome. This protocol has been previously used to induce delayed-onset muscle soreness (DOMS) [18].

### 2.3. Convalescence: Astragalosides and Placebo Supplement

Each subject performed the 7-day trial in a randomized order, with the astragalosides supplementation or placebo-control trial separated by at least 1 week. Astragalosides (in total 4 mg per day; Sun Ten Pharmaceutical Co., New Taipei, Taiwan) or placebo capsules were ingested immediately after the muscle damage protocol and consumed in the morning for 7 days after the exercise. According to the manufacturer, 1.746 to 4.365 mg daily of bioactive astragalosides, as dry powder, is recommended for use by humans. Placebo capsules consisted of hydroxypropyl methylcellulose, and subjects could not distinguish this from the astragalosides.

All subjects were required to keep their diet as constant as possible during the study. Dietary intake was recorded for 3 days in the week of the intervention program to assess potential changes in daily food intake that might have occurred during the intervention period. In the second intervention program, the subjects were instructed to consume the same compositional diet as in the first intervention, thereby minimizing the impact of differences in food intake.

### 2.4. Quantitative Analysis of Astragalosides Dry Powder in Capsules

Astragalosides were purchased as dry powder from Sun Ten pharmaceutical factory (New Taipei, Taiwan). Internal standards (astragaloside I, II, III, and IV) were purchased from ChromaDex Inc. (Irvine, CA, USA). Samples and standards were prepared in methanol and filtered through a 0.45 μm filter, and then injected to HPLC for assay. The injection volume was 5 μL. The HPLC separation was performed with a Phenomenex Kinetix C18 column (150 × 4.6 mm, 2.6 μm, 100 Å) by gradient elution. The mobile phase consisted of an initial composition of 5% acetonitrile (HPLC grade, Fairfield, OH, USA) and 95% water (Milli-Q, Milford, MA, USA), with gradient elution at a flow rate of 0.9 mL/min at 40 °C. Astragalosides analysis was performed by high-performance liquid chromatography with a charged aerosol detector (HPLC/Corona-CAD).

### 2.5. Assessment of Muscle Pain

As DOMS may not be felt without mechanical stimulus, standardized stretching or muscle-contraction protocol is recommended to assess perceived muscle soreness of the quadriceps [19]. Participants performed 10 repetitions of flexing and extending at the knee, throughout the entire range of motion, on a seated leg extension machine using a load corresponding to each subject’s 75% 1 RM. The severity of the quadriceps femoris muscle pain was monitored by a visual analogue scale (VAS) score after the standardized stimulus protocol [20]. The VAS was a continuous line of 100 mm on which 0 indicated “no pain” and 100 represented “extreme pain”.

### 2.6. Biochemical Analysis

Blood samples were collected at the following intervals: before and after eccentric exercise, and at 2 hr, and 1, 2, 3, 5, and 7 days of the recovery period. Blood samples were drawn from the cephalic vein and centrifuged at 1000× *g* for 10 min at 4 °C. Samples were analyzed for human IGF-I, IGF-II, creatine kinase (CK), lactate dehydrogenase (LDH), myoglobin (Mb), IL-6, and TNF-α. Serum CK, LDH, and Mb levels were measured by a DT-60 II analyzer (Johnson & Johnson, Rochester, NY). Serum IGF-I, IGF-II, IL-6, and TNF-α were analyzed by enzyme-linked immunosorbent assay (ELISA) (R&D Systems, Minneapolis, MN, USA), in triplicate.

### 2.7. Assessment of Muscular Strength

Peak isokinetic torque (PIT) of the knee extensors was measured on an isokinetic dynamometer (Biodex Medical Systems 3 pro, Biodex Medical Inc., Shirley, NY, USA) before the muscle damage protocol, and at 1, 2, 3, 5, and 7 days of the recovery period. After a warm-up, subjects were seated on an isokinetic dynamometer with restraining straps securing the trunk, pelvis, and thigh. The axis of the knee was aligned with the central axis of the dynamometer, and the lower extremity was secured to a resistance lever arm. Before PIT testing, the lower limb was gravity corrected by the dynamometer, and the range of motion was set between 60° and 30° (0° =horizontal, 90° = vertical) of knee flexion. During testing, subjects were given visual feedback for torque and encouraged to “kick out” as hard as possible for maximal torque. All tests were performed in triplicate with a 1 min rest interval between efforts.

### 2.8. Muscle Oxygenation Monitoring

Muscle oxygenation was monitored immediately after eccentric exercise and at 7 days of the recovery period. The participants’ left vastus lateralis was noninvasively attached to the portable PortaMon continuous wave near-infrared spectroscopy (NIRS) system (Artinis Medical Systems, Zetten, The Netherlands) with two wavelengths, and participants performed 10 repetitions of flexion and extension of the knee, at 75% 1 RM, to monitor muscle oxygenation and to calculate the tissue oxygen saturation index (TSI). Muscle oxygenation was measured by relative changes in micromolar (µmol/L) concentrations of oxyhemoglobin (O_2_Hb), deoxyhemoglobin (HHb), and total hemoglobin (tHb) [21]. Emitted near-infrared light of two wavelengths of 760 and 850 nm was mainly absorbed separately by HbO_2_ and HHb in small arterioles, capillaries, and venules within the muscle. With these concentrations, the TSI was calculated. NIRS measurement was reliably used to assess the changes in local muscle O_2_ extraction during exercise [22].

### 2.9. Statistical Analyses

All data are presented as means ± SD. Statistical analysis was performed using SPSS version 14.0 software (SPSS, Inc., Chicago, IL, USA), with the significance level set at *p* < 0.05. Data were analyzed for treatment (astragalosides and placebo) and timepoints (Pre, Post, 2 h, 1, 2, 3, 5, and 7 days) using two-way repeated ANOVA. The data for all biomarkers, muscle soreness, and muscle strength were analyzed to detect time effects of muscle repair by using one-way ANOVA. In case of significant *F* ratios, post hoc comparisons were performed through Tukey’s HSD test, and the Bonferroni alpha-level correction was applied to explore the different effects of time under the two treatment conditions.

## 3. Results

### 3.1. Subjects’ Characteristics

Table 1 illustrates the subjects’ characteristics for this study.

### 3.2. Astragalosides Quantification

**Figure 1:**
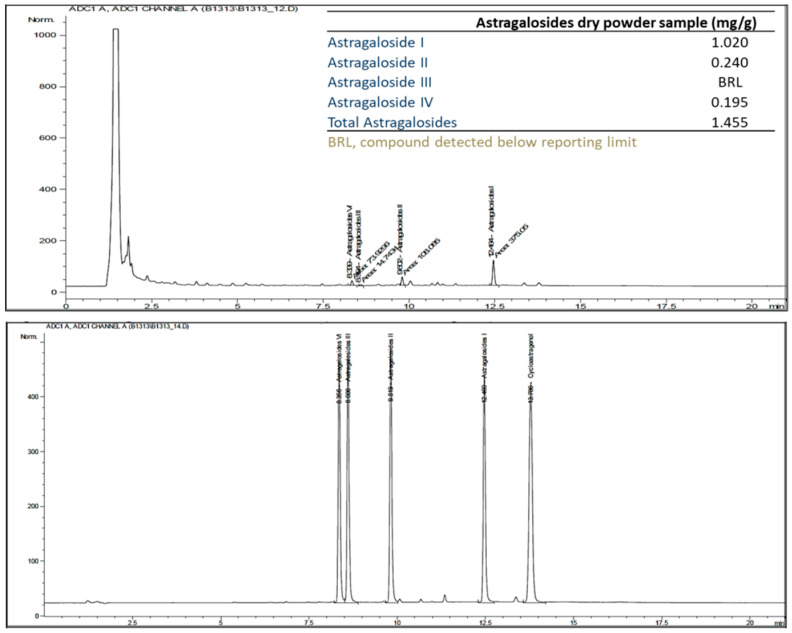
HPLC/Corona-CAD chromatogram profile of astragalosides in the astragalosides capsules. The composition of the astragalosides compound in the astragalosides capsules is indicated in the upper right table.

The results of the HPLC/Corona-CAD analysis revealed that the main compounds in the dry astragalosides powder were astragaloside I, II, III, and IV (Figure 1).

### 3.3. Cellular Markers of Muscle Damage

**Figure 2:**
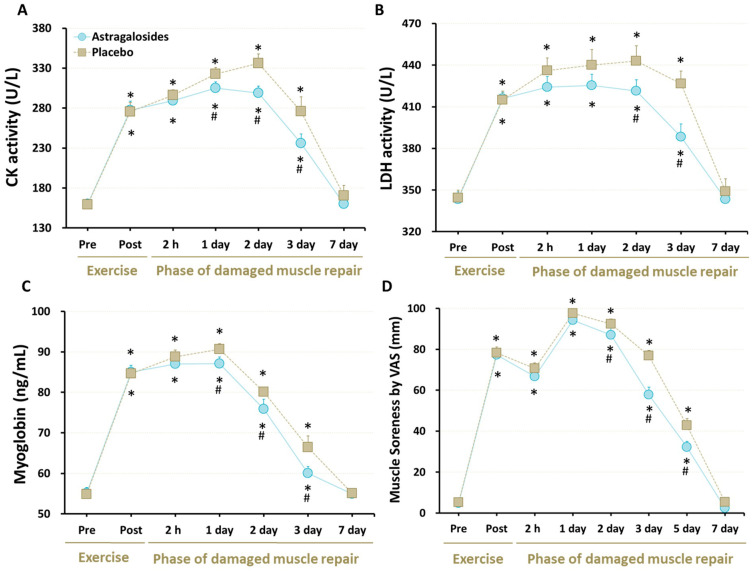
Effect of astragalosides supplementation on muscle-damage markers. (**A**) Serum creatine kinase (CK) activity; (**B**) serum lactate dehydrogenase (LDH) activities; (**C**) serum myoglobin levels; (**D**) muscle soreness. The levels of creatine kinase activity, lactate dehydrogenase activity, and myoglobin were measured before and after eccentric exercise, and at 2 hr, and 1, 2, 3, and 7 days of the recovery period. The subjects’ severity of quadriceps femoris muscle pain was assessed before and after eccentric knee-extension exercise, and at 2 h, and 1, 2, 3, 5, and 7 days of the recovery period. Values are expressed as means ± SD (*n* =11). * *p* < 0.05 vs. pre-exercise for the same group. # *p* < 0.05 vs. placebo for the same time.

At baseline, all of the cellular markers for muscle damage were similar for astragalosides and placebo trials (all *p* > 0.05). As expected, serum levels of CK, LDH, and Mb were elevated significantly in response to eccentric exercise in both trials (*p* < 0.05, Figure 2A–C), showing that this eccentric exercise protocol was sufficient to induce destruction of normal myofibrils. However, astragalosides supplementation resulted in lower CK and Mb levels from days 1 to 3 following eccentric exercise, when compared to the placebo group, while astragalosides supplementation resulted in lower LDH activity from days 2 to 3 following eccentric exercise, when compared to placebo.

### 3.4. Muscle Soreness

All subjects experienced an increase in perceived muscle soreness at days 1 and 2 following eccentric exercise, compared with baseline (*p* < 0.05, Figure 2D). Although perceived muscle soreness returned to baseline at day 7, astragalosides supplementation reduced perceived muscle soreness at days 2, 3, and 5 following eccentric exercise, compared with placebo (interaction between supplementation and time points: *p* < 0.05).

### 3.5. Inflammation Responses and Regeneration Regulatory Factors

**Figure 3:**
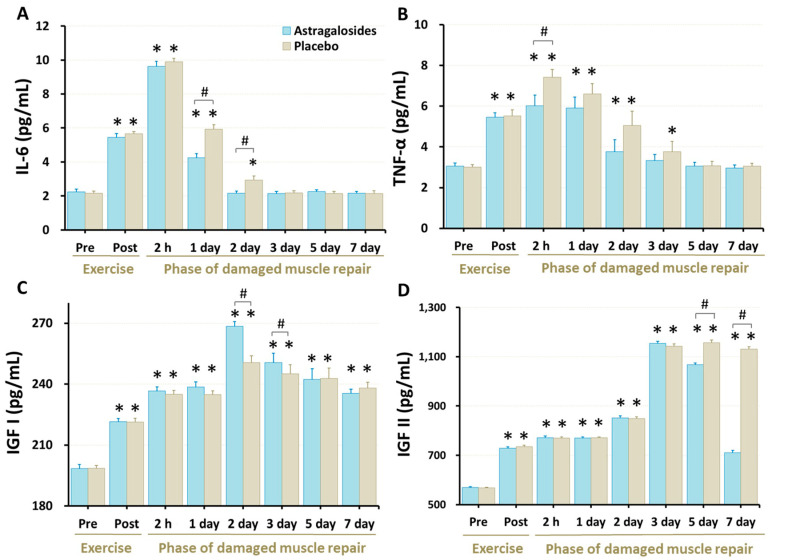
Effect of astragalosides supplementation on serum levels of (**A**) interleukin-6 (IL-6), (**B**) tumor necrosis factor-alpha (TNF-α), (**C**) insulin-like growth factor I (IGF-I), and (**D**) insulin-like growth factor II (IGF-II). The levels of IL-6, TNF-α, IGF-I, and IGF-II were measured before and after eccentric exercise, and at 2 h, 1, 2, 3, 5, and 7 days of the recovery period. Values are expressed as means ± SD (*n* = 11). * *p* < 0.05 vs. pre-exercise for the same group. # *p* < 0.05 vs. placebo for the same time.

Our data revealed that the serum IL-6 and TNF-α concentrations were higher immediately following eccentric exercise (all *p* < 0.05) and returned to baseline 2–3 days after eccentric exercise (all *p* < 0.05; Figure 3A,B). Astragalosides supplementation was associated with lower serum IL-6 levels at days 1 and 2 after exercise when compared with placebo (*p* < 0.05, Figure 3). Similarly, astragalosides supplementation was associated with lower TNF-α levels than placebo 2 h after eccentric exercise (*p* < 0.05). Furthermore, following astragalosides supplementation, both IL-6 and TNF- α returned to baseline one day earlier than placebo.

Serum IGF-I and IGF-II concentrations were higher following the eccentric exercise (all *p* < 0.05, Figure 3C,D). Astragalosides supplementation resulted in higher IGF-I than placebo at days 2 and 3, and in lower IGF-II concentration than placebo at days 5 and 7 (*p* < 0.05).

### 3.6. Recovery of Muscle Respiratory Capacity

**Figure 4:**
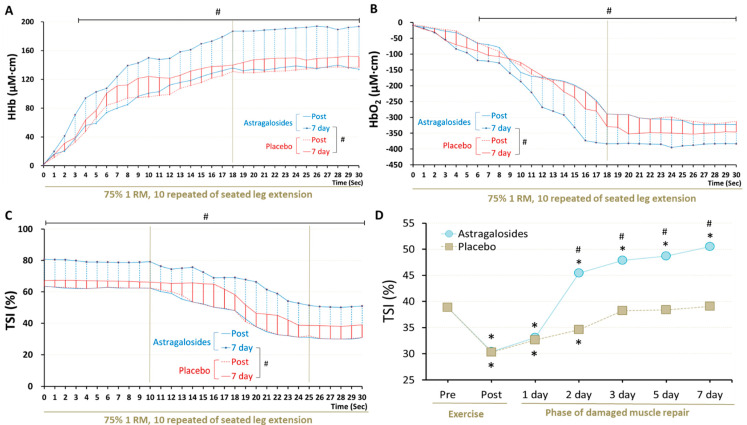
Effect of astragalosides supplementation on recovery of muscle oxygenation capacity after muscle damage. (**A**) The vastus lateralis muscle deoxyhemoglobin (HHb), (**B**) oxyhemoglobin (O_2_Hb), (**C**) tissue oxygen saturation index (TSI), and (**D**) TSI at last 5 s of seated leg extension were monitored under 10 repititions of 75% 1 RM seated leg extension. # indicates the main significant effect for the group (*p* < 0.05). * *p* < 0.05 vs. pre-exercise for the same group.

Astragalosides supplementation and placebo resulted in different muscle oxygenation responses after knee extension exercise at day 7 (*p* < 0.05, Figure 4). Muscle HHb reached a plateau at 18 s (the value remained approximately 187 to 193.5 μM·cm) and was higher following astragalosides supplementation compared with placebo from 1 s to the end of the exercise at day 7 (Figure 4A). HbO_2_ declined rapidly from 0 to 18 s during exercise, displaying a basin from 18 to 30 s (values remained approximately −383 to −390 μM·cm, Figure 4B). Muscle HHb increased throughout exercise, while HbO_2_ dropped significantly after 7 days of astragalosides supplementation. We observed a significant increase in the final calculated TSI of working muscles after 7 days of astragalosides supplementation (Figure 4C).

### 3.7. Recovery of Muscle Function

**Figure 5:**
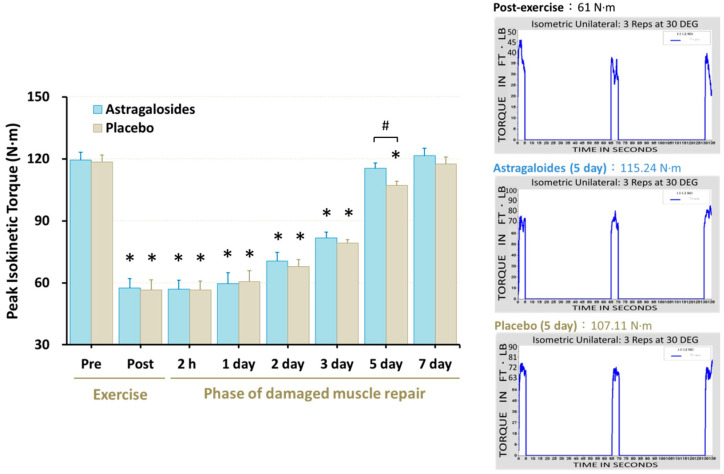
Effect of astragalosides supplementation on muscular force recovery after muscle damage. Peak isokinetic torque was measured before and after eccentric exercise, and at 2 h, 1, 2, 3, 5, and 7 days of the recovery period. Values are expressed as means ± SD (*n* = 11). * *p* < 0.05 vs. pre-exercise for the same group. # *p* < 0.05 vs. placebo for the same time. The right panel shows representative peak isokinetic torque.

Significant reduction of maximal isometric quadriceps muscle force was observed immediately following muscle damage, remaining at the end of day 3 (*p* < 0.05, Figure 5). However, astragalosides supplementation resulted in an early recovery of maximal isometric quadriceps muscle force when compared to placebo at day 5 (*p* < 0.05, Figure 5).

## 4. Discussion

It has not previously been investigated whether astragalosides supplementation could potentially alter the duration of the inflammatory response and promote faster skeletal muscle repair after eccentric exercise. We therefore sought to determine whether astragalosides supplementation can shorten the duration of the inflammatory period and promote quicker recovery of skeletal muscle and skeletal muscular strength. Five key findings emerge from this study: following muscle damage, astragalosides supplementation can (1) rapidly eliminate the accumulation of muscle-damage markers such as serum CK, LDH, and Mb; (2) shorten the duration of exercise-induced inflammation; (3) promote the release of muscle regenerative hormone IGF-1; (4) enhance muscle oxygenation in repaired skeletal muscle; (5) increase the rate of force recovery in damage muscle. These findings agree with our study hypothesis. Collectively, this study indicates that 7 days of astragalosides supplementation is an effective way to promote faster recovery of the skeletal muscle function following eccentric exercise-induced muscle damage.

### 4.1. Astragalosides Supplementation and Chemical Analysis

From the structural point of view, the difference between isomers I and II lies in the difference of glycosyl configuration, and the glycosyl junction sites of III and IV are different. The same mother nucleus and different post glycosylation modifications may endow the four astragalosides with different physiological activities.

Generally, astragalosides exhibit a wide range of biological activities, such as antioxidant and neuroprotective effects and immune regulation [23]. The diversity of glycosyl configuration and junction sites greatly affects the functions of astragalosides. To date, previous publications have often focused on astragaloside IV. It has been suggested that its anti-oxidant, anti-inflammatory, and anti-apoptotic properties are the reasons for neuroprotection, liver protection, anti-cancer, and anti-diabetes properties. These pharmacological effects are associated with multiple signaling pathways, such as the AMPK signaling pathway, Akt/PDE3B signaling pathway, and Raf-MEK-ERK pathway [24,25,26]. Meanwhile, comparatively few studies have focused on astragaloside I and astragaloside II. Astragaloside I stimulates osteoblast differentiation through the Wnt/β-catenin signaling pathway [27], and can also combine levistilide A and calycosin for anti-liver fibrosis effects [28]. Astragaloside II can promote intestinal epithelial repair and trigger T-cell activation through activating the mTOR pathway and CD45 protein tyrosine phosphatase, respectively [29,30]. Unfortunately, Astragaloside III is a neglected substance without relevant research.

### 4.2. Astragalosides Supplementation and Muscle Damage Markers

Immediately after the eccentric excise protocol and in the days following (Figure 2), we observed a sustained increase of CK, LDH, and Mb in both groups, indicating evidence of acute muscle damage. We successfully illustrated that these markers of muscle damage were lower up to 3 days after exercise with astragalosides supplementation, compared with the placebo group. A plausible physiological mechanism could be due to the protective effects of astragalosides supplementation on muscle tissue to reduce secondary damage caused by neutrophil and monocyte activity. This would mean that less CK, LDH, and Mb leaks out in the days after exercise as secondary damage to the tissue is reduced. This is also the plausible biological mechanism of why muscle soreness (Figure 2D) and inflammation were lower following astragalosides supplementation (Figure 3A,B). While we cannot explain why the lactate dehydrogenase activity was slightly above the normal pre-exercise range, we are confident this was not from the inflammatory response, as all participants avoided any strength training or high-intensity training for one week prior to participating in the study.

### 4.3. Astragalosides Supplementation and Inflammatory Responses

We observed higher IL-6 and TNF-α secretion immediately after overloaded eccentric exercise (Figure 3A,B), consistent with previous studies [18,31]. This elevation of pro-inflammatory markers could represent an early stage of muscle regeneration, and coincide with an influx of neutrophils and macrophages into the injured skeletal muscle. Subsequently, pro-inflammatory cytokines (TNF-α and IL-6) can be suppressed by macrophages and provoke an anabolic signaling cascade, which is thought to be particularly important for the outcome of the regeneration process [32,33]. 

Recent publications have revealed that macrophages play a key role in the early repair process of skeletal muscles, and contribute to skeletal muscle myogenesis. When skeletal muscle injury occurs, macrophages transition from an inflammatory phenotype to a regenerative phenotype for normal muscular repair [34]. During the macrophage regeneration phase, macrophages secrete high levels of IGF-1 [35] to support myogenesis, followed by low levels of TNF-α [36], which fosters myogenic differentiation. From our experimental data (Figure 2B), it can be inferred that astragalosides supplementation may also be involved in immunoregulation through macrophage activity, as TNF-α levels were significantly lower 2 h after astragalosides supplementation.

Additionally, we found that astragalosides supplementation greatly suppressed the release of pro-inflammatory cytokines such as IL-6 and TNF-α, and thus facilitated faster restoration of skeletal muscular strength following eccentric exercise-induced muscle damage (Figure 5). Based on the previous view that astragalosides are good anti-inflammatory and immunomodulator regulators [23], we believe that astragalosides play a crucial role in balancing between pro-inflammatory and anti-inflammatory cytokines to attenuate excessive inflammatory reaction. Additionally, Wang et al. [37] revealed that astragalosides have a protective effect on muscle cells, especially astragaloside IV which was shown to significantly suppress the release of interleukin-8 in diaphragmatic muscle cells.

### 4.4. Astragalosides Supplementation and Early Stage of Muscular Repair

IGF-I has been shown to regulate differentiation in damaged muscle fibers by activating myogenic regulatory factors [4,38]. After eccentric exercise, the myofiber and the satellite cells upregulate the gene expression of IGF-I, resulting in greater production of IGF-I between 24–72 h post-exercise [38]. Furthermore, endocrine and autocrine as well as paracrine IGF mechanisms have been shown to mediate muscle development and repair, an early response critical in the regenerative process [39,40,41]. In line with this, our results revealed that for both treatments IGF-I in reached its peak value following 48 h post-exercise (Figure 3C). Our data also revealed that 7 days of astragalosides supplementation resulted in higher serum IGF-I levels, indicating that astragalosides supplementation may promote faster skeletal muscle repair after exercise-induced muscle damage. Astragalosides supplementation improves serum IGF-I levels, possibly due to the fact that astragalosides are an IGF-I activator which can effectively upregulate IGF-1 expression [42]. However, the presence of astragalosides accelerates the return of serum IGF-II levels to baseline (Figure 3D, days 5 and 7). In contrast to IGF-I, little is known about how serum IGF-II levels are regulated during myogenesis. The underlying mechanisms remain unclear, making the interpretation of this differential phenomenon complex, thus warranting further investigation. Based on the available evidence, current consensus holds that IGF-II plays a role in the later stages of myoblast differentiation, while allowing myotube formation [43,44]. Erbay et al. [45] revealed that the autocrine IGF-II transcription required for skeletal myocyte differentiation is regulated by mTOR and nutritional amino acids. Indeed, we previously demonstrated that *Astragalus membranaceus* is a nutritional activator promoting lean muscle anabolism through increasing the Akt/mTOR signaling pathway [16]. This may partially explain why lower IGF-II concentration was observed following astragalosides supplementation, compared with the placebo, during the later stages of muscular recovery. Our results implicate a potential role for astragalosides in regulating circulating IGF levels during skeletal muscle development.

### 4.5. Astragalosides Supplementation and Muscle Strength Performance

There is a temporal association between the extent of muscle strength loss after exercise and the time required for skeletal muscular force to recover to normal. Skeletal muscular force loss by <20% immediately after eccentric exercise is usually restored within 2–3 days post-exercise [46,47]. By contrast, when muscular force loss is ~50% immediately after eccentric exercise, it requires at least 1 week of recovery, and leukocytes accumulate in the skeletal muscle during the 24 h after exercise, while circulating interleukin levels increase [48,49]. In line with this, our results for the time course of changes showed that muscular strength, DOMS, and blood CK, LDH, Mb, and IL-6 levels recovered slowly in the days after intense eccentric exercise in the placebo group. In contrast, muscle soreness, biomarkers of muscle damage, and inflammatory response were efficiently restored after astragalosides supplementation, which could be a plausible explanation for why muscular strength returned to baseline 2 days earlier than the placebo group following eccentric exercise (Figure 5).

### 4.6. Astragalosides Supplementation and Muscle Respiratory Functional Performance

When muscle function does not fully return to normal, the performance of working muscles declines and manifests as a mismatch between intramuscular oxygen supply and demand during exercise [10]. We continuously monitored hemodynamic parameters and the oxygenation response throughout a standardized 30-s exercise protocol, using NIRS technology. NIRS revealed changes in microvascular concentrations of oxyhemoglobin and deoxyhemoglobin, reflecting the dynamic balance between muscle oxygen delivery and extraction in the underlying tissue [50,51]. Moreover, NIRS also calculated tissue oxygen saturation, reflecting the balance between intramuscular oxygen supply and demand [52]. In the present study, we found a favourable effect of astragalosides supplements on recovery of damaged muscle. One previous study reported that 16 days of amino acid supplemention enhanced the muscle oxygenation index of the vastus lateralis in moderate-intensity exercise testing [53,54]. Xu et al. [55] observed that sarcoplasmic reticulum Ca-ATPase gene expression in hypoxic-injured cardiomyocytes increased after astragalosides treatment, indicating a beneficial effect on muscle respiratory capacity. These reports are compatible with our results indicating that astragalosides supplementation could enhance skeletal muscle oxygenation and thus promote quicker recovery of the injured skeletal muscles.

## 5. Conclusions

In conclusion, based on our findings, astragalosides supplementation can be considered an ergogenic aid that eliminates intracellular accumulation of muscle-damage biomarkers, and thus shortens the duration of intramuscular inflammation while promoting faster muscle repair processes and the restoration of muscular strength.

## Figures and Tables

**Table 1:** Subjects’ Characteristics.

Characteristics	(*n* = 11)	
Age (year)	23 ± 0.9	
Body weight (Kg)	73.4 ± 1.9	
Height (m)	1.75 ± 0.02	
Body mass index (kg/m^2^)	24.0 ± 0.7	
Leg volume (liters)	7.5 ± 0.3	
1RM leg press (kg)	217.7 ± 7.0	
1RM leg extension (kg)	120.2 ± 3.4	
	Astragalosides period	Placebo period
Total energy (Kcal)	1987 ± 65	1984 ± 77
Carbohydrate (% of energy)	51.4 ± 2.3	51.6 ± 2.2
Fat (% of energy)	30.8 ± 1.4	30.6 ± 1.3
Protein (% of energy)	17.8 ± 1.2	17.8 ± 1.4

All values are presented as mean ± SD. Abbreviations: 1 RM = subject’s one repetition maximum.

## Data Availability

Not applicable.
